# Sensing Leakage of Electrolytes from Magnesium Batteries Enabled by Natural AIEgens

**DOI:** 10.3390/ijms231810440

**Published:** 2022-09-09

**Authors:** Yingxiang Zhai, Jiguo Zhang, Jian Li, Shouxin Liu, Zhijun Chen, Shujun Li

**Affiliations:** Key Laboratory of Biobased Material Science and Technology of Ministry of Education, Northeast Forestry University, Hexing Road 26, Harbin 150040, China

**Keywords:** laccaic acid, aggregation-induced emission, smart film, detect of Mg^2+^

## Abstract

The potential for leakage of liquid electrolytes from magnesium (Mg) batteries represents a large hurdle to future application. Despite this, there are no efficient sensing technologies to detect the leakage of liquid electrolytes. Here, we developed a sensor using laccaic acid (L-AIEgen), a naturally occurring aggregation-induced emission luminogen (AIEgens) isolated from the beetle Laccifer lacca. L-AIEgen showed good selectivity and sensitivity for Mg^2+^, a universal component of electrolytes in Mg batteries. Using L-AIEgen, we then produced a smart film (L-AIE-F) that was able to sense leakage of electrolytes from Mg batteries. L-AIE-F showed a strong “turn-on” AIE-active fluorescence at the leakage point of electrolyte from model Mg batteries. To the best of our knowledge, this is the first time that AIE technology has been used to sense the leakage of electrolytes.

## 1. Introduction

**Figure 1:**
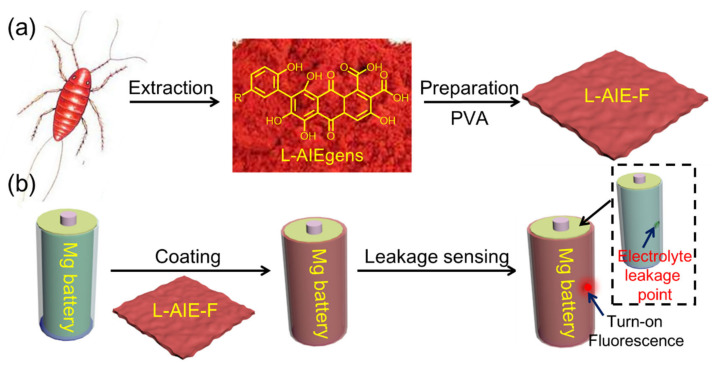
Schematic illustrations of the (**a**) preparation of L-AIE-F and (**b**) fluorescence sensing of electrolyte leakage from Mg batteries.

Magnesium (Mg) metal is an attractive anode material for rechargeable batteries, because it has a low reduction potential (−2.37 V vs. normal hydrogen electrode), a higher volumetric capacity than lithium and, unlike lithium, does not form dendrites during plating-stripping cycles [1,2,3,4,5]. Although lithium-ion batteries (LIBs) are widely used in portable electronic devices and electric vehicles due to the fact of their high energy density and long service life, the rapid consumption of LIBs is not sustainable due to the limited mineral resources of inorganic electrodes [6,7]. These issues limit the penetration of LIB technology into the large-scale energy storage market [8]. Since Mg is also inexpensive, highly abundant and environmentally benign, the Mg-metal rechargeable battery has long been viewed as a safe and low-cost alternative to the popular lithium-ion battery [9,10,11,12]. One of main drawbacks with Mg-metal batteries is the possible leakage of electrolytes, which are typically Grignard reagent, organoborate, borohydride, magnesium aluminate chloride complex, or Mg(TFSI)_2_-based solutions [13]. The leakage of these liquid electrolytes can cause many problems such as the corrosion of the metal casing of the battery [14], heavy metal ions in the electrolyte can cause environmental pollution [15], and fire and explosions can occur due to the leakage of flammable electrolytes [16]. Although there is thus an urgent need to develop a sensitive method for detecting leakage of electrolytes from Mg batteries, little attention has been paid to this problem. Fluorescence technologies are a good option for sensing, since they can provide fast, sensitive, and accurate analyses of guest species [17,18,19,20,21,22,23,24]. Fluorescent probes show a “turn-on” or “turn-off” fluorescence response to characteristic signal compounds in the analyzed guests [25,26,27,28,29,30,31,32,33,34,35]. The Mg^2+^ ion is one of the most abundant divalent ions, and they play a vital role in many chemical, biological, and environmental processes. In recent years, a variety of fluorescent probe have been developed by different research groups for Mg^2+^ detection. Suzuki et al. reported two new Mg^2+^ fluorescence imaging probes, KMG-20-AM and KMG-27-AM, both of which have a β-hydroxycarboxylate group and an aromatic amino group combined with a conjugated π-electron system, which will bring great changes in the fluorescence spectrum after forming a Mg^2+^ complex [36]. Ceroni and coworkers synthesized a hexathiobenzene molecule carrying six terpyridine units, and after adding of Mg^2+^ ions to the molecule in THF solution, metal-bridged crosslinking supramolecular polymer aggregates were formed, resulting in the observable turn-on phosphorescence [37]. Tang et al. report an efficient and convenient procedure for detecting Mg^2+^ with an AIE-active fluorescence probe in acetonitrile; this receptor showed a sensitive response to the addition of Mg^2+^ with enhanced fluorescence aggregation [38]. Among these probes, those based on aggregation-induced emission luminogen (AIEgens) are particularly attractive. AIE-active fluorescence was first reported by Tang’s group in 2001 [39]. Unlike traditional fluorescent chromophores, AIEgens become more emissive when aggregated [40,41,42,43,44]. Since AIEgen-based probes remain highly emissive in the aggregated or solid state, and they are readily portable and can conveniently be used as solids to sense guests, without the need for dissolution or other sophisticated pretreatments [45]. Motivated by these properties, here, we developed a novel naturally occurring AIEgen, laccaic acid (L-AIEgen), which can be extracted from the beetle Laccifer lacca. Compared with synthetic AIEgens, naturally occurring AIEgens are biocompatible, easily prepared and cheap [46,47,48]. The L-AIEgen showed a sensitive “turn-on” fluorescence to Mg^2+^. As a result, L-AIEgen was mixed with polyvinyl alcohol (PVA) to prepare composite films (L-AIE-F) for sensing leakage of electrolytes from Mg batteries (Figure 1). L-AIE-F showed a sensitive “turn-on” fluorescence when exposed to leakage of electrolytes (LX-144, 0.4 M (MgPhCl)_2_-AlCl_3_) from model Mg batteries and the detection limit was low at ~3.26 mmol.

## 2. Results and Discussion

**Figure 2:**
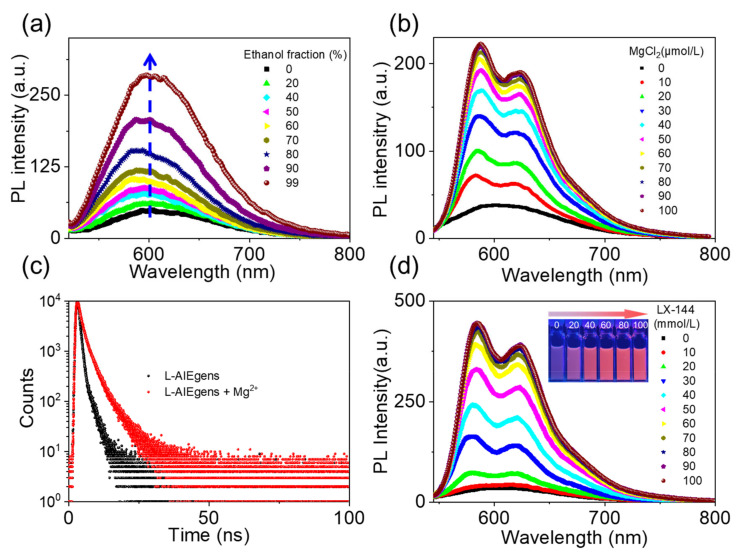
(**a**) Changes in the fluorescence of L-AIEgen in aqueous solution upon the addition of ethanol, with an excitation wavelength = 500 nm; (**b**) changes in the fluorescence of L-AIEgen (10 ppm) in ethanol solution upon the addition of MgCl_2_, with an excitation wavelength = 520 nm; (**c**) fluorescence lifetime of L-AIEgen in ethanol solution (10 ppm) in the presence and absence of Mg^2+^ (10 ppm), with an excitation wavelength = 520 nm; (**d**) changes in the fluorescence of L-AIEgen (10 ppm) in ethanol solution upon the addition of different volumes of LX-144 (0.4 m). PL Intensity (a.u.) = photoluminescence intensity (arbitrary units), with an excitation wavelength = 520 nm.

The fluorescence of L-AIEgen in aqueous solution was very weak, but it intensified upon the addition of ethanol (Figure 2a and Table 1). When the fraction of ethanol reached 99%, the fluorescence intensity increased approximately six-fold, indicating AIE-active fluorescence of L-AIEgen. The absorption spectra of L-AIEgen were studied, and a red shift in the absorption peak was observed when ethanol was added, indicating the formation of J-aggregates (Figure S1) [48]. The addition of MgCl_2_ to an aqueous solution of L-AIEgen also enhanced the AIE fluorescence in a concentration-dependent manner (Figure 2b) and increased the fluorescence lifetime from 2.5 to 4.1 ns (Figure 2c). A wide variety of other cations were used to assess the selectivity of L-AIEgen, and none of these appreciably enhanced fluorescence (Figure S2). With the addition of Mg^2+^ ions into the L-AIEgen solution, coordination between Mg^2+^ and L-AIEgen occurred, resulting in fluorescence enhancement and a UV-vis absorption red shift (Figure S3). It might be attributed to the magnesium, which is preferred for forming a six-coordinated octahedral geometry by using N and O as ligands [49], the coordination of lone pairs of electrons on the N or O donor atoms to the Mg^2+^ sites, thereby stabilizing the excited state relative to the ground state, leading to longer wavelength absorption [50]. Encouraged by this high selectivity for Mg^2+^, we next evaluated the ability of L-AIEgen to sense LX-144, a typical Mg^2+^-containing electrolyte used in Mg batteries. The addition of LX-144 to a solution of L-AIEgen increased the fluorescence intensity in a concentration-dependent manner (Figure 2d). We compared the increase in the fluorescence intensity of L-AIEgen when the concentration of LX-144 and Mg^2+^ were the same, and we found that the fluorescence intensity of the LX-144 was not as good as Mg^2+^. This may be because the Mg in LX-144 exists in the form of (MgPhCl)_2_-AlCl_3_ complex, and its contact reaction with L-AIEgen was not as good as Mg^2+^, resulting in the fluorescence intensity of LX-144 being not as good as Mg^2+^ (Figure S4). In short, the spectra of L-AIEgen in the presence of increasing concentrations of LX-144 were similar to those in the presence of increasing concentrations of MgCl_2_, suggesting that fluorescence enhancement of L-AIEgen can be attributed to its reaction with Mg^2+^. All of these results demonstrate that L-AIEgen is, as expected, sensitive to LX-144, and that the sensitivity can be attributed to its reaction with Mg^2+^.

**Figure 3:**
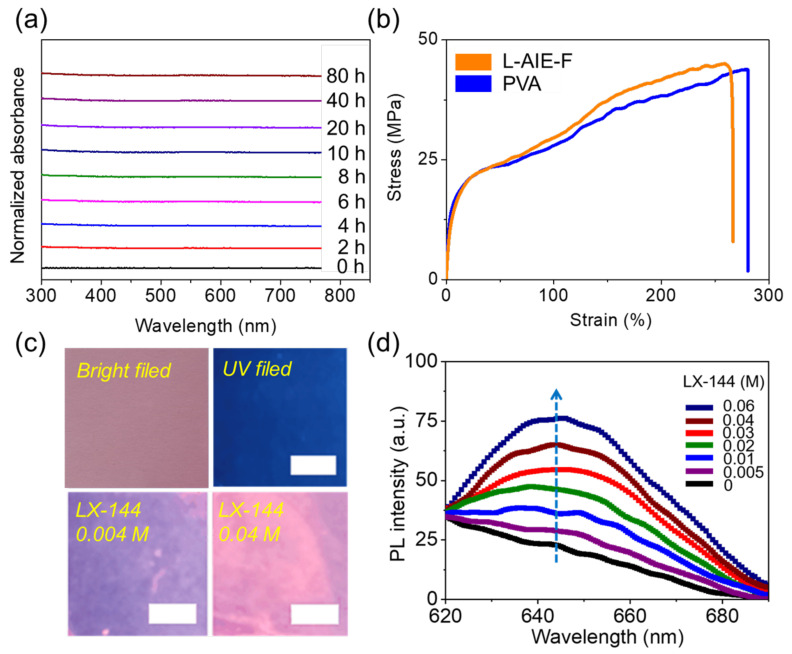
(**a**) In situ measurement of the absorbance of THF (10 mL) in the presence of L-AIE-F (2 × 2 cm) for different periods of time; (**b**) tensile strength of L-AIE-F and PVA; (**c**) images of L-AIE-F under bright field (**upper left**), UV field (**upper right**), UV field in the presence of 0.05 M LX-144 (**lower left**), and UV field in the presence of 0.4 M LX-144 (**lower right**); (**d**) fluorescence emission of L-AIE-F in the presence of different concentrations of LX-144, with an excitation wavelength = 365 nm.

L-AIE-F was prepared by mixing L-AIEgen and PVA in aqueous solution, and its basic physical performance as a film was investigated. The SEM images (Figure S5) showed that laccaic acid was evenly distributed in the PVA matrix. Both laccaic acid and PVA molecules are rich in hydroxyl groups, which results in their high polarity and good compatibility. The appearance of L-AIE-F is shown in Figure S6. L-AIE-F can maintain a stable state in the ambient state, and it is still very stable after being placed in the air for 80 h. Its fluorescence spectrum is shown in Figure S7. Migration experiments using THF, and monitored with UV-Vis spectroscopy, showed that no L-AIEgen had leached into the THF, even after contact for 80 h (Figure 3a and Figure S8). Meanwhile, we also performed migration experiments using other solvents (i.e., water, ethanol, and ethyl ether) and monitored them using UV-Vis spectroscopy. After 80 h of exposure, no L-AIEgen was leached into these solvents (Figure S9), indicating that L-AIE-F was not only stable in THF, but also in water, ethanol, and ethyl ether. L-AIEgen was thus stably fixed in the PVA matrix, likely because of the hydrogen bonds between the hydroxyl groups of PVA and the phenolic groups of L-AIEgen. The mechanical performance of L-AIE-F was investigated next. The tensile strength and elongation at break were 44 MPa and 256%, respectively (Figure 3b and Figure S10). The tensile strength and elongation of PVA were 42 MPa and 269%, respectively (Figure 3b and Figure S10), showing that incorporation of L-AIEgen did not appreciably alter the mechanical strength of the PVA matrix, and the increase in the tensile strength of L-AIE-F (42 MPa to 44 MPa) indicated that there may be hydrogen bonds between the hydroxyl groups of PVA and the phenolic groups of L-AIEgen, which enhanced the interaction between PVA and L-AIEgen [51,52]. L-AIE-F was thus stable and had good mechanical performance. At the same time, we also measured the transmittance of L-AIE-F (Figure S11). After adding 0.1% wt laccaic acid to PVA, L-AIE-F still had a good transmittance, and the transmittance was still greater than 70% in the visible region (400–800 nm). The fluorescence of L-AIE-F was then measured in the presence of electrolyte containing Mg^2+^. L-AIE-F showed a concentration-dependent enhancement of fluorescence upon the addition of LX-144 (Figure 3c). Upon addition of Mg^2+^, the maximum fluorescence emission of L-AIE-F was at ~645 nm, representing a bathochromic shift compared with the fluorescence of L-AIEgen and LX-144 in solution (Figure 3d). The red shift in fluorescence might be attributable to the molecular J-type aggregation of L-AIEgen in the PVA matrix. Therefore, the UV absorption spectra of L-AIEgen and L-AIE-F were measured, and it was found that when L-AIEgen was in the PVA solute, the absorption peak showed an obvious red shift (Figure S12), from 488 to 523 nm, indicating the possible formation of J-aggregates [53]. Preliminary experiments were next carried out to investigate the sensitivity of the fluorescence emission of L-AIE-F to LX-144. The fluorescence emission of L-AIE-F showed marked enhancement upon the addition of LX-144 (Figure 3c,d). The relationship fitted the linear equation: y = 0.9x + 25 (R = 0.99), where the fluorescence is 645 nm measured at a given Mg^2+^ concentration (0–60 mm), and x is the concentration of Mg^2+^ added (Figure S13). The detection limit (3 s/K, s = standard deviation of the blank signal, K = 0.9) was ~3.26 mmol These results unambiguously confirmed that L-AIE-F was sensitive to LX-144, an electrolyte commonly used in Mg batteries.

**Figure 4:**
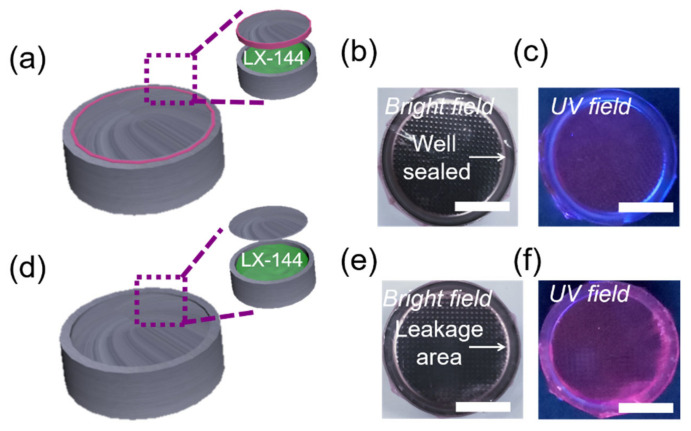
(**a**) Schematic illustration of well-sealed Mg battery model; images of the Mg battery model coated with L-AIE-F (**b**) in bright field and (**c**) upon UV irradiation (365 nm), scale bar = 0.5 cm; (**d**) schematic illustration of a leaking Mg battery model; images of a leaking Mg battery model coated with L-AIE-F (**e**) in bright field and (**f**) upon UV irradiation (365 nm), scale bar = 0.5 cm.

Encouraged by the electrolyte-triggered enhancement of AIE, we next tested whether L-AIE-F could be used for fluorescence sensing of electrolyte leakage. To mimic Mg batteries, LX-144 electrolyte was placed in coin cell shells, with and without sealing rings, (Figure 4a,d), and the shells were then coated with L-AIE-F (Figure 4b,e). The fluorescence of L-AIE-F did not change noticeably when it was coated on the outside of coin cell shells with sealing rings (Figure 4c), but a strong enhancement in the fluorescence was observed when it was coated on the outside of shells without sealing rings (Figure 4f). These results unequivocally confirm that L-AIE-F could be used to sense leakage of electrolytes from Mg^2+^ batteries.

## 3. Materials and Methods

### 3.1. Materials

Laccaic acid was obtained from the Research Institute of Resources Insects, Chinese Academy of Forestry, Beijing, China. Poly (vinyl alcohol) (PVA, average degree of polymerization = 1750 ± 50) was purchased from Sigma-Aldrich, Shanghai, China. All other reagents and solvents were purchased from Merck Life Science Co., Ltd., Shanghai, China, or Shanghai Aladdin Bio-Chem Technology Co., Ltd., Shanghai, China. LX-144 electrolyte (0.4 M (MgPhCl)_2_-AlCl_3_ in THF) was purchased from Alibaba, Hangzhou, China.

### 3.2. Characterization

UV-Vis absorption spectra of L-AIEgen were recorded over the range 200–800 nm using a TU-1901 ultraviolet-visible double-beam spectrophotometer (Persee General Instrument Co., Ltd., Beijing, China). Photoluminescence (PL) was measured using a Fluo-max 4 spectrofluorometer (Horiba Scientific, Piscataway, NJ, USA). Tensile strength and elongation at break of L-AIE-F were measured using a UTM-2203 electromechanical universal testing machine (Suns Technology Stock Co., Ltd., Shenzhen, China). All measurements were performed at room temperature.

### 3.3. Preparation of L-AIEgen

Solution A: 10 mg laccaic acid was dissolved in 10.0 mL water to form 1 mg/mL laccaic acid solution.

### 3.4. Preparation of L-AIE-F

PVA (2.5 g) was dissolved in deionized water (50 mL), the solution was stirred magnetically for 2 h at 90 °C, and lac dye (2.5 mg) was then added. After stirring for a further 10 min, the mixture was poured onto a glass plate and dried naturally to give L-AIE-F. The films were dried at 30 °C and 50% humidity for 72 h before testing.

### 3.5. Sensing Electrolyte Leakage

LX-144 was placed in coin cell shells, with and without a sealing ring, to model intact and leaking Mg batteries. Then cut the L-AIE-F into a 2 × 2 cm square to completely coated onto these Mg battery models. After shaking the button battery, the electrolyte LX-144 in the Mg batteries without the sealing ring will leak. When the L-AIE-F film covering the outside of the Mg batteries contact with the leaked LX-144, the fluorescence of the film will increase accordingly. Electrolyte leakage was detected upon 365 nm UV irradiation.

## 4. Conclusions

In summary, we prepared a composite film (L-AIE-F) based on a naturally occurring AIEgen (L-AIEgen). In the presence of Mg^2+^, L-AIE-F showed a sensitive enhancement of AIE. This property allowed specific in situ detection of electrolyte leakage from a model Mg battery, demonstrating that L-AIE-F can be used practically for this purpose. Since L-AIE-F can be easily and cheaply prepared, it can be produced on a large scale and used commercially. In the future, L-AIE-F might be processed as a smart coating for Mg batteries, which can sense the leakage of electrolytes in situ. Additionally, following our strategy, AIEgen-based probes for other cations, such as Li^+^ and Fe^3+^ [54,55,56,57], might also be prepared as smart coatings to sense electrolyte leakage from Li or Fe batteries.

## Figures and Tables

**Table 1:** Preparation of laccaic acid solutions with different ethanol fraction.

Ethanol Fraction (%)	Solution A (mL)	Water (mL)	Ethanol (mL)
0	0.1	9.9	0
20	0.1	7.9	2
40	0.1	5.9	4
50	0.1	4.9	5
60	0.1	3.9	6
70	0.1	2.9	7
80	0.1	1.9	8
90	0.1	0.9	9
99	0.1	0	9.9

## Data Availability

The data presented in this study are available upon request from the corresponding author.

## Supplementary Materials

The following supporting information can be downloaded at: https://www.mdpi.com/article/10.3390/ijms231810440/s1.

## References

[1] Shterenberg I., Salama M., Gofer Y., Levi E., Aurbach D. (2014). The challenge of developing rechargeable magnesium batteries. MRS Bull..

[2] Tutusaus O., Mohtadi R., Arthur T.S., Mizuno F., Nelson E.G., Sevryugina Y.V. (2015). An efficient halogen-free electrolyte for use in rechargeable magnesium batteries. Angew. Chem. Int. Ed..

[3] Barile C.J., Barile E.C., Zavadil K.R., Nuzzo R.G., Gewirth A.A. (2014). Electrolytic conditioning of a magnesium aluminum chloride complex for reversible magnesium deposition. J. Phys. Chem. C.

[4] Tian H., Gao T., Li X., Wang X., Luo C., Fan X., Yang C., Suo L., Ma Z., Han W. (2017). High power rechargeable magnesium/iodine battery chemistry. Nat. Commun..

[5] Canepa P., Jayaraman S., Cheng L., Rajput N.N., Richards W.D., Gautam G.S., Curtiss L.A., Persson K.A., Ceder G. (2015). Elucidating the structure of the magnesium aluminum chloride complex electrolyte for magnesium-ion batteries. Energy Environ. Sci..

[6] Lu D., Liu H., Huang T., Xu Z., Ma L., Yang P., Qiang P., Zhang F., Wu D. (2018). Magnesium ion based organic secondary batteries. J. Mater. Chem. A.

[7] Song M., Niu J., Gao H., Kou T., Wang Z., Zhang Z. (2020). Phase engineering in lead-bismuth system for advanced magnesium ion batteries. J. Mater. Chem. A.

[8] Tarascon J.M., Armand M. (2001). Issues and challenges facing rechargeable lithium batteries. Nature.

[9] Kim H.S., Arthur T.S., Allred G.D., Zajicek J., Newman J.G., Rodnyansky A.E., Oliver A.G., Boggess W.C., Muldoon J. (2011). Structure and compatibility of a magnesium electrolyte with a sulphur cathode. Nat. Commun..

[10] Crowther O., West A.C. (2008). Effect of electrolyte composition on lithium dendrite growth. J. Electrochem. Soc..

[11] Xu K. (2004). Nonaqueous liquid electrolytes for lithium-based rechargeable batteries. Chem. Rev..

[12] Aurbach D., Cohen Y., Moshkovich M. (2001). The study of reversible magnesium deposition by in situ scanning tunneling microscopy. Electrochem. Solid-State Lett..

[13] Attias R., Salama M., Hirsch B., Goffer Y., Aurbach D. (2018). Anode-electrolyte interfaces in secondary magnesium batteries. Joule.

[14] Rodriguez J., Chenoy L., Roobroeck A., Godet S., Olivier M.G. (2016). Effect of the electrolyte pH on the corrosion mechanisms of Zn-Mg coated steel. Corros. Sci..

[15] Zalyhina V., Cheprasova V., Romanovski V. (2021). Pigments from spent ammonium chloride zinc plating electrolytes. J. Chem. Technol. Biotechnol..

[16] Henriksen M., Vaagsaether K., Lundberg J., Forseth S., Bjerketvedt D. (2019). Explosion characteristics for Li-ion battery electrolytes at elevated temperatures. J. Hazard. Mater..

[17] Shi L., Gao X., Yuan W., Xu L., Deng H., Wu C., Yang J., Jin X., Zhang C., Zhu X. (2018). Endoplasmic Reticulum-Targeted Fluorescent Nanodot with Large Stokes Shift for Vesicular Transport Monitoring and Long-Term Bioimaging. Small.

[18] Chen X., Liu X., Lei J., Xu L., Zhao Z., Kausar F., Xie X., Zhu X., Zhang Y., Yuan W.Z. (2018). Synthesis, clustering-triggered emission, explosive detection and cell imaging of nonaromatic polyurethanes. Mol. Syst. Des. Eng..

[19] Yuan W.Z., Wang Q., Dou X., Chen X., Zhao Z., Wang S., Wang Y., Sui K., Tan Y., Gong Y. (2019). Reevaluating the Protein Emission: Remarkable Visible Luminescence and Emissive Mechanism. Angew. Chem. Int. Ed..

[20] Dong S., Xu J., Jia T., Xu M., Zhong C., Yang G., Li J., Yang D., He F., Gai S. (2019). Upconversion-mediated ZnFe_2_O_4_ nanoplatform for NIR-enhanced chemodynamic and photodynamic therapy. Chem. Sci..

[21] Feng L., He F., Dai Y., Liu B., Yang G., Gai S., Niu N., Lv R., Li C., Yang P. (2017). A versatile near infrared light triggered dual-photosensitizer for synchronous bioimaging and photodynamic therapy. ACS Appl. Mater. Interfaces.

[22] He F., Feng L., Yang P., Liu B., Gai S., Yang G., Dai Y., Lin J. (2016). Enhanced up/down-conversion luminescence and heat: Simultaneously achieving in one single core-shell structure for multimodal imaging guided therapy. Biomaterials.

[23] Sun W., Li S., Häupler B., Liu J., Jin S., Steffen W., Schubert U.S., Butt H.J., Liang X.J., Wu S. (2017). An amphiphilic ruthenium polymetallodrug for combined photodynamic therapy and photochemotherapy in vivo. Adv. Mater..

[24] Sun W., Parowatkin M., Steffen W., Butt H.J., Mailänder V., Wu S.J. (2016). Ruthenium-Containing Block Copolymer Assemblies: Red-Light-Responsive Metallopolymers with Tunable Nanostructures for Enhanced Cellular Uptake and Anticancer Phototherapy. Adv. Healthc. Mater..

[25] Saengsrichan A., Saikate C., Silasana P., Khemthong P., Wanmolee W., Phanthasri J., Youngjan S., Posoknistakul P., Ratchahat S., Laosiripojana N. (2022). The Role of N and S Doping on Photoluminescent Characteristics of Carbon Dots from Palm Bunches for Fluorimetric Sensing of Fe^3+^ Ion. Int. J. Mol. Sci..

[26] Fang X., Yan D. (2018). White-light emission and tunable room temperature phosphorescence of dibenzothiophene. Sci. China Chem..

[27] Li X., Baryshnikov G., Deng C., Bao X., Wu B., Zhou Y., Ågren H., Zhu L. (2019). A three-dimensional ratiometric sensing strategy on unimolecular fluorescence-thermally activated delayed fluorescence dual emission. Nat. Commun..

[28] Zhou Y., Baryshnikov G., Li X., Zhu M., Ågren H., Zhu L. (2018). Anti-Kasha’s rule emissive switching induced by intermolecular H-bonding. Chem. Mater..

[29] Wu Y., Xie Y., Zhang Q., Tian H., Zhu W., Li A.D. (2014). Quantitative photoswitching in bis (dithiazole) ethene enables modulation of light for encoding optical signals. Angew. Chem. Int. Ed..

[30] Li D., Hu W., Wang J., Zhang Q., Cao X.-M., Ma X., Tian H. (2018). White-light emission from a single organic compound with unique self-folded conformation and multistimuli responsiveness. Chem. Sci..

[31] Li Y., Liu C., Chen M., An Y., Zheng Y., Tian H., Shi R., He X., Lin X. (2022). Solvent-Free Preparation of Tannic Acid Carbon Dots for Selective Detection of Ni^2+^ in the Environment. Int. J. Mol. Sci..

[32] Tian Z., Yan Q., Feng L., Deng S., Wang C., Cui J., Wang C., Zhang Z., James T.D., Ma X. (2019). A far-red fluorescent probe for sensing laccase in fungi and its application in developing an effective biocatalyst for the biosynthesis of antituberculous dicoumarin. Chem. Commun..

[33] Wu L., Wang Y., James T.D., Jia N., Huang C. (2018). A hemicyanine based ratiometric fluorescence probe for mapping lysosomal pH during heat stroke in living cells. Chem. Commun..

[34] Chen G., Shao W., Valiev R.R., Ohulchanskyy T.Y., He G.S., Ågren H., Prasad P.N. (2016). Efficient Broadband Upconversion of Near-Infrared Light in Dye-Sensitized Core/Shell Nanocrystals. Adv. Opt. Mater..

[35] Shao W., Chen G., Kuzmin A., Kutscher H.L., Pliss A., Ohulchanskyy T.Y., Prasad P.N. (2016). Tunable narrow band emissions from dye-sensitized core/shell/shell nanocrystals in the second near-infrared biological window. J. Am. Chem. Soc..

[36] Suzuki Y., Komatsu H., Ikeda T., Saito N., Araki S., Citterio D., Hisamoto H., Kitamura Y., Kubota T., Nakagawa J. (2002). Design and synthesis of Mg^2+^-selective fluoroionophores based on a coumarin derivative and application for Mg^2+^ measurement in a living cell. Anal. Chem..

[37] Fermi A., Bergamini G., Roy M., Gingras M., Ceroni P. (2014). Turn-on Phosphorescence by Metal Coordination to a Multivalent Terpyridine Ligand: A New Paradigm for Luminescent Sensors. J. Am. Chem. Soc..

[38] Bian Y.-J., Wang L.-Q., Cao F.-X., Tang L.-J. (2016). A Simple Fluorescence Probe Based on Aggregation-Induced Emission (AIE) Property for the Detection of Mg^2+^ Ions. J. Fluoresc..

[39] Luo J., Xie Z., Lam J.W., Cheng L., Chen H., Qiu C., Kwok H.S., Zhan X., Liu Y., Zhu D. (2001). Aggregation-induced emission of 1-methyl-1, 2, 3, 4, 5-pentaphenylsilole. Chem. Commun..

[40] Chen Y., Lam J.W., Kwok R.T., Liu B., Tang B.Z. (2019). Aggregation-induced emission: Fundamental understanding and future developments. Mater. Horiz..

[41] Hong Y., Lam J.W.Y., Tang B.Z. (2011). Aggregation-induced emission. Chem. Soc. Rev..

[42] Mei J., Hong Y., Lam J.W., Qin A., Tang Y., Tang B.Z. (2014). Aggregation-induced emission: The whole is more brilliant than the parts. Adv. Mater..

[43] Wang J., Lin X., Shu T., Su L., Liang F., Zhang X. (2019). Self-assembly of metal nanoclusters for aggregation-induced emission. Int. J. Mol. Sci..

[44] Feng G., Liu B. (2018). Aggregation-induced emission (AIE) dots: Emerging theranostic nanolights. Acc. Chem. Res..

[45] He T., Wang H., Chen Z., Liu S., Li J., Li S. (2018). Natural Quercetin AIEgen Composite Film with Antibacterial and Antioxidant Properties for in Situ Sensing of Al^3+^ Residues in Food, Detecting Food Spoilage, and Extending Food Storage Times. ACS Appl. Bio Mater..

[46] Ma Z., Liu C., Niu N., Chen Z., Li S., Liu S., Li J. (2018). Seeking brightness from nature: J-aggregation-induced emission in cellulolytic enzyme lignin nanoparticles. ACS Sustain. Chem. Eng..

[47] Wang P., Liu C., Tang W., Ren S., Chen Z., Guo Y., Rostamian R., Zhao S., Li J., Liu S. (2019). Molecular Glue Strategy: Large-Scale Conversion of Clustering-Induced Emission Luminogen to Carbon Dots. ACS Appl. Mater. Interfaces.

[48] He T., Niu N., Chen Z., Li S., Liu S., Li J. (2018). Novel quercetin aggregation-induced emission luminogen (AIEgen) with excited-state intramolecular proton transfer for in vivo bioimaging. Adv. Funct. Mater..

[49] Case D.R., Zubieta J., Doyle R.P. (2020). The Coordination Chemistry of Bio-Relevant Ligands and Their Magnesium Complexes. Molecules.

[50] Chairat M., Rattanaphani V., Bremner J.B., Rattanaphani S., Perkins D.F.J.D. (2004). An absorption spectroscopic investigation of the interaction of lac dyes with metal ions. Dyes Pigments.

[51] Xie W., Bao Q., Liu Y., Wen H., Wang Q. (2021). Hydrogen Bond Association to Prepare Flame Retardant Polyvinyl Alcohol Film with High Performance. ACS Appl. Mater. Interfaces.

[52] Song P., Wang H. (2020). High-Performance Polymeric Materials through Hydrogen-Bond Cross-Linking. Adv. Mater..

[53] Cao W., Sletten E.M. (2018). Fluorescent cyanine dye J-Aggregates in the fluorous phase. J. Am. Chem. Soc..

[54] Yuan H., Peng H.J., Li B.Q., Xie J., Kong L., Zhao M., Chen X., Huang J.Q., Zhang Q. (2019). Conductive and Catalytic Triple-Phase Interfaces Enabling Uniform Nucleation in High-Rate Lithium-Sulfur Batteries. Adv. Energy Mater..

[55] Tu S.B., Chen X., Zhao X.X., Cheng M.R., Xiong P.X., He Y.W., Zhang Q., Xu Y. (2018). A Polysulfide-Immobilizing Polymer Retards the Shuttling of Polysulfide Intermediates in Lithium-Sulfur Batteries. Adv. Mater..

[56] Yan C., Cheng X.B., Yao Y.X., Shen X., Li B.Q., Li W.J., Zhang R., Huang J.Q., Li H., Zhang Q. (2018). An Armored Mixed Conductor Interphase on a Dendrite-Free Lithium-Metal Anode. Adv. Mater..

[57] Li B.Q., Zhang S.Y., Kong L., Peng H.J., Zhang Q. (2018). Porphyrin Organic Framework Hollow Spheres and Their Applications in Lithium-Sulfur Batteries. Adv. Mater..

