# Supplementary material for: Sensing Leakage of Electrolytes from Magnesium Batteries Enabled by Natural AIEgens
Source: Int J Mol Sci. 2022 Sep 9;23(18):10440. doi: 10.3390/ijms231810440 (PMC9499604; doi:10.3390/ijms231810440)
Supplement: Supplementary file 1 [file ijms-23-10440-s001.zip › ijms-1886019-supplementary.pdf]

Supporting Information

Sensing Leakage of Electrolytes from Magnesium Batteries Enabled by Natural AIEgens

Yingxiang Zhai, Jiguo Zhang, Jian Li, Shouxin Liu, Zhijun Chen and Shujun Li**

Key Laboratory of Bio-based Material Science and Technology of Ministry of Education, Northeast Forestry University, Hexing Road 26, Harbin 150040, China

*Email: chenzhijun@nefu.edu.cn (Z.C.); lishujun@nefu.edu.cn (S.L.)

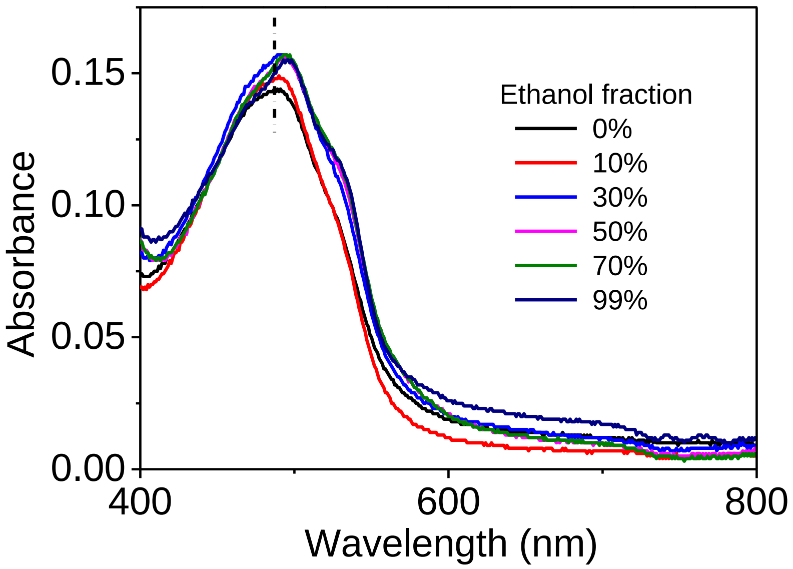

Figure S1. UV-Vis spectra of L-AIEgen (10 ppm) in mixtures of water and ethanol.

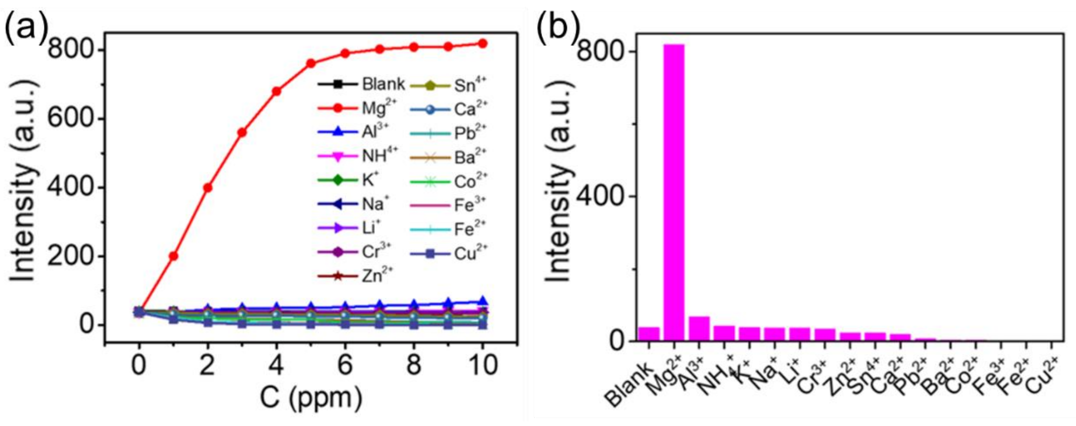

Figure S2. Fluorescence emission of L-AIEgen (10 ppm, 2.5 mL) in the presence of different cations. Ex = 520 nm, Em = 586 nm. (a) Fluorescence emission titrations (0-10 ppm) of L-AIEgen in the presence of different cations. (b) The fluorescence comparison of L-AIEgen at 586 nm upon adding 10 ppm of different cations.

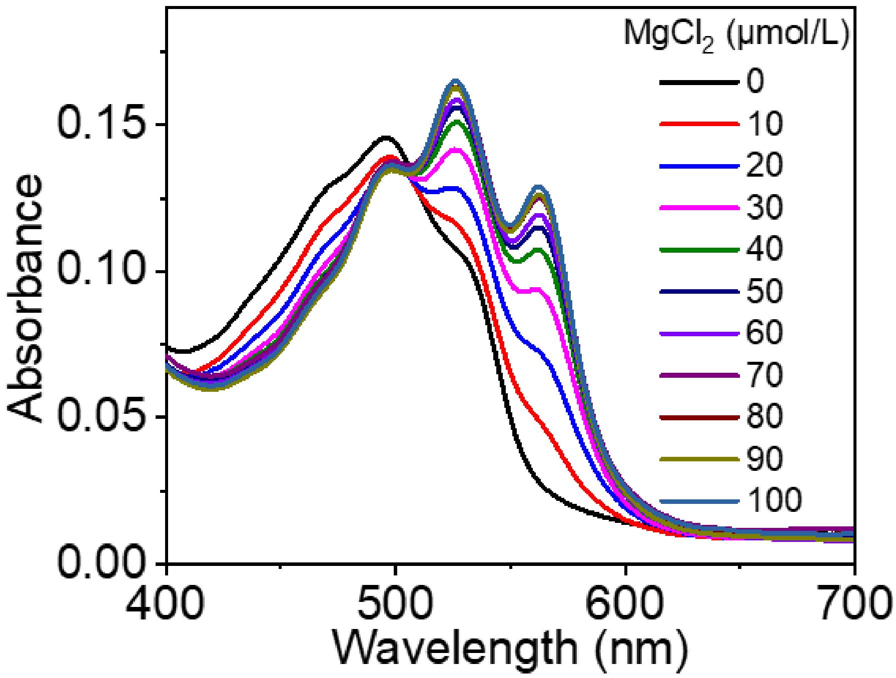

Figure S3. Changes in absorbance of L-AIEgen (10 ppm) in ethanol solution upon addition of $MgCl_2$.

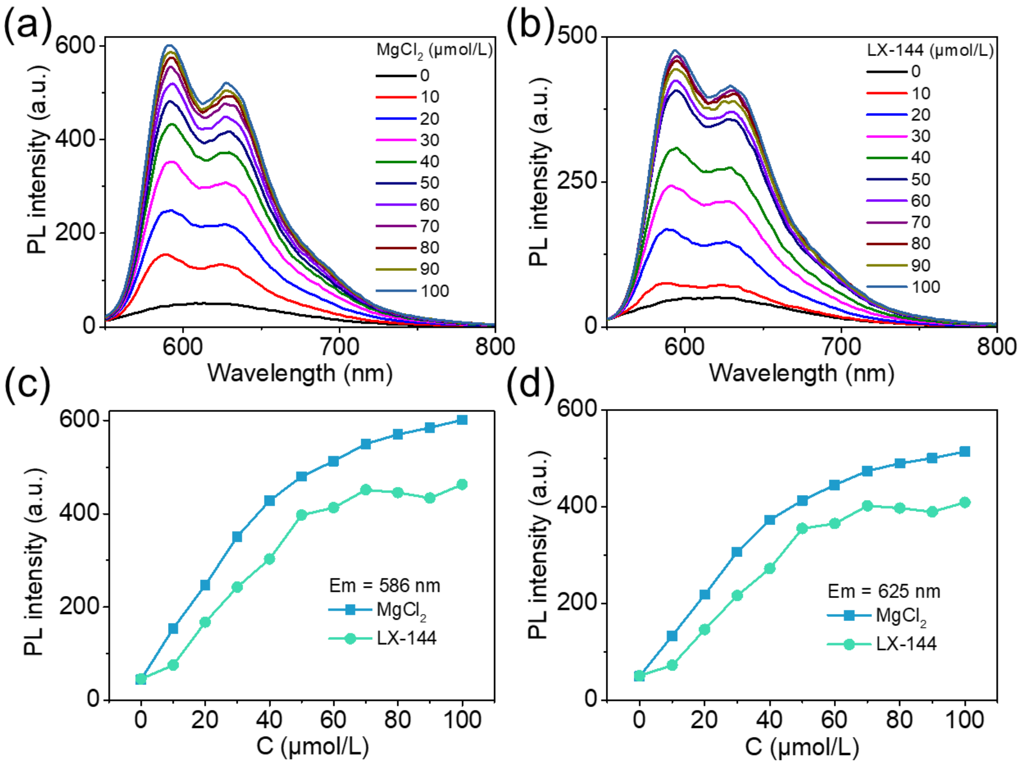

Figure S4. The effect of the same concentration of Mg^{2+} and LX-144 on the fluorescence intensity of L-AIEgen. (a) Changes in fluorescence of L-AIEgen (10 ppm) in ethanol solution upon addition of $MgCl_2$ (0-100 μmol/L), Ex = 520 nm; (b) Changes in fluorescence of L-AIEgen (10 ppm) in ethanol solution upon addition of LX-144 (0-100 μmol/L), Ex = 520 nm; (c) Comparison of L-AIEgen fluorescence intensity after adding Mg^{2+} and LX-144, Em = 586 nm; (d) Comparison of L-AIEgen fluorescence intensity after adding Mg^{2+} and LX-144, Em = 625 nm.

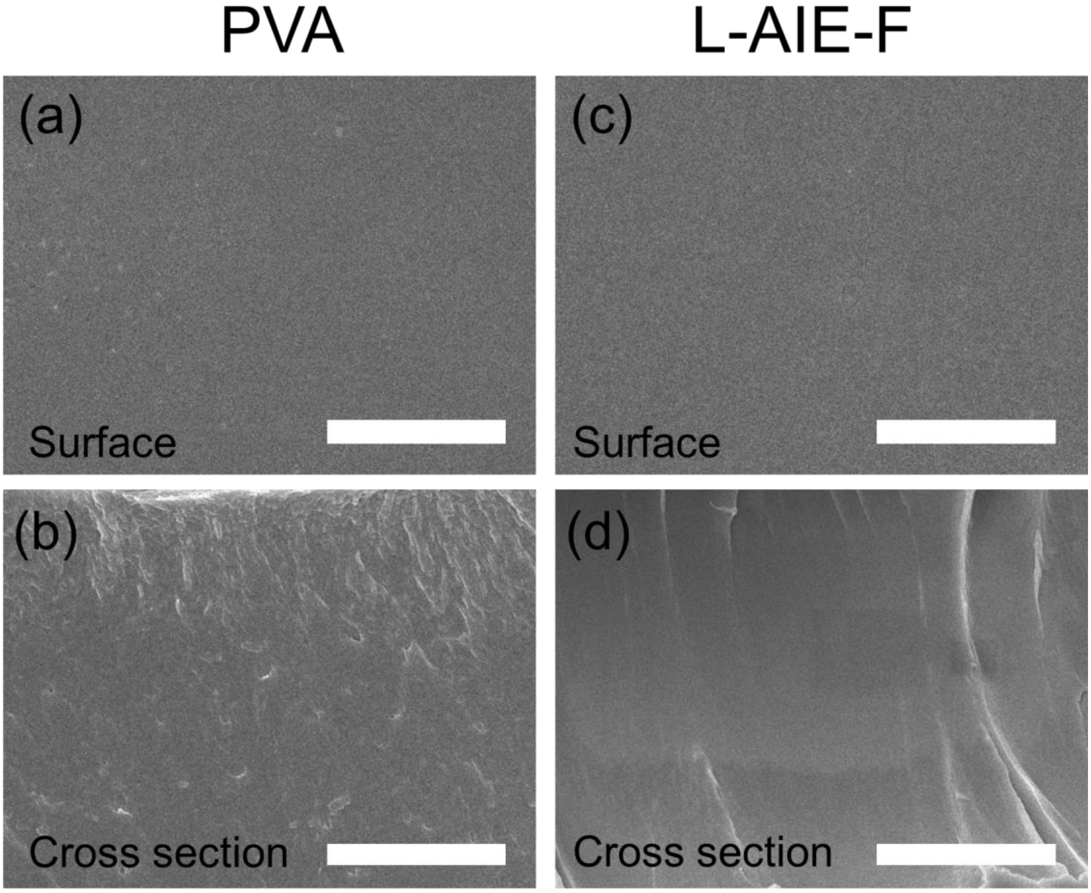

Figure S5. SEM images of the surface of (a) PVA and (c) L-AIE-F and cross section of (b) PVA and (d) L-AIE-F, scale bar = 5 μm.

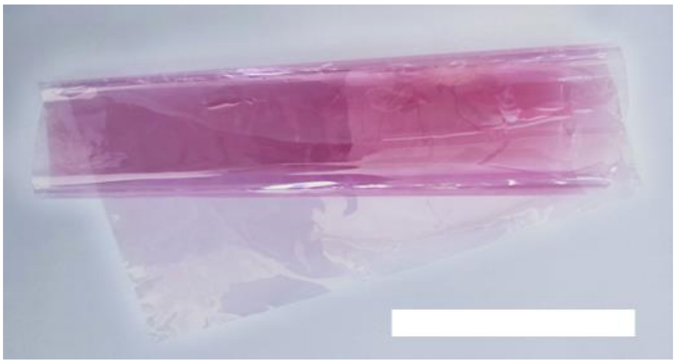

Figure S6. Image of L-AIE-F, scale bar = 5 cm.

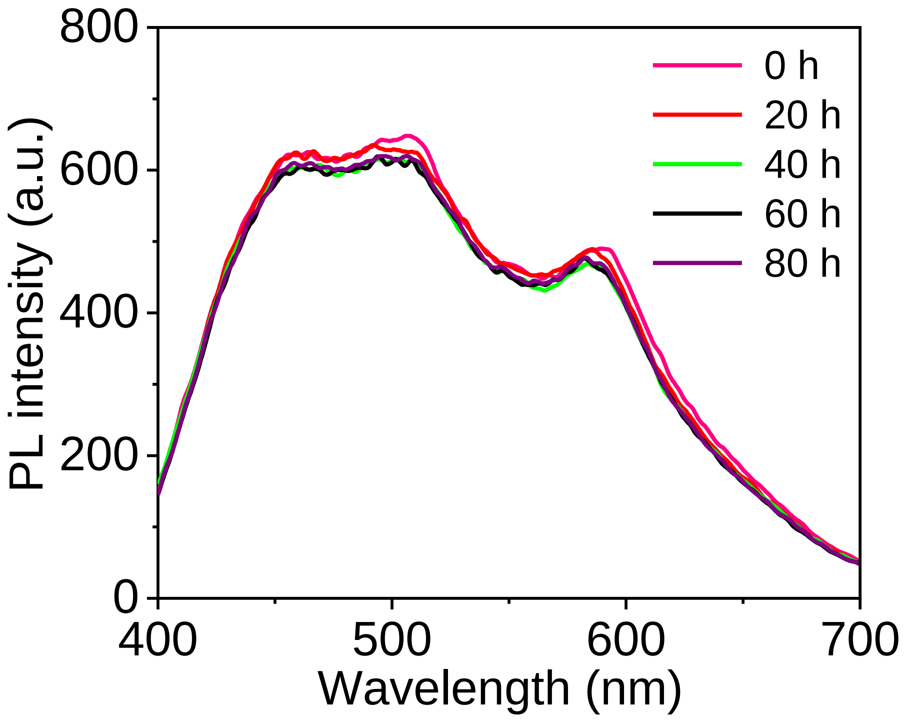

Figure S7. The fluorescence spectrum of L-AIE-F in the ambient state, excitation wavelength = 365 nm.

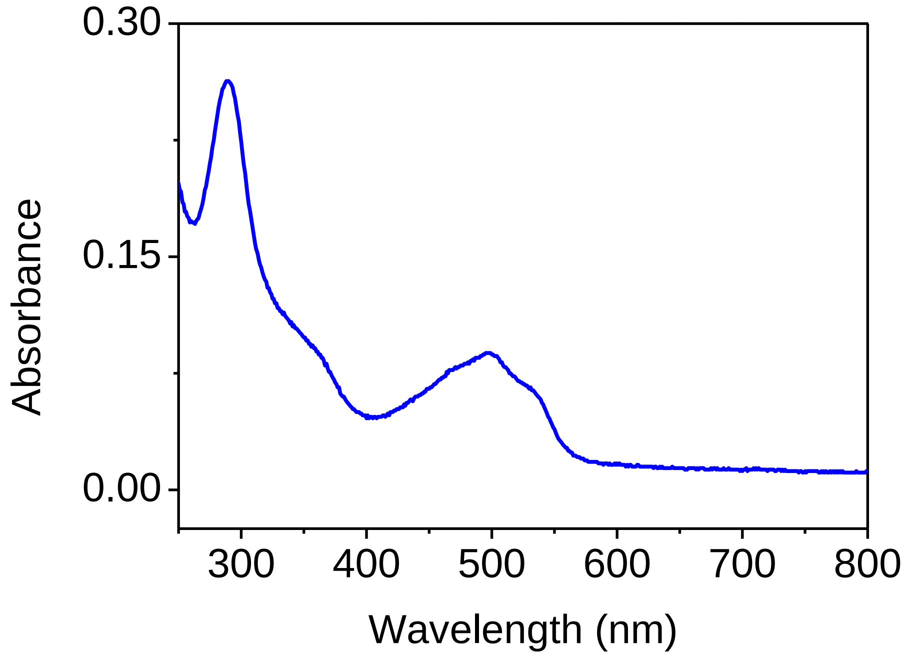

Figure S8. Absorbance of L-AIEgen in THF (10 ppm).

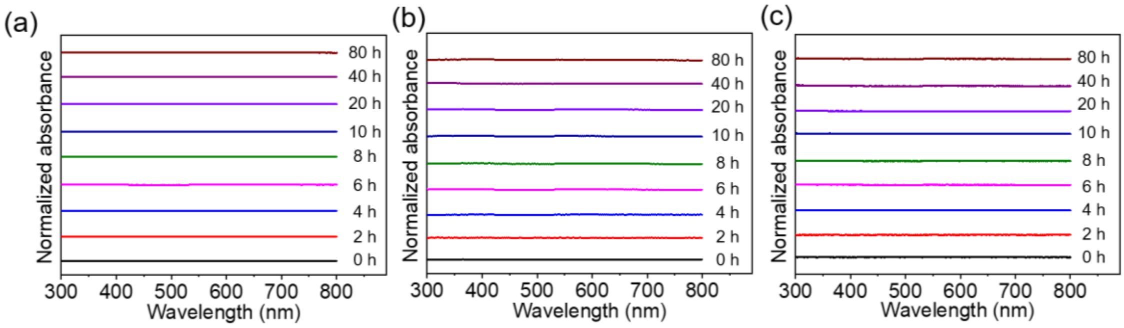

Figure S9. In situ measurement of absorbance of water (a), ethanol (b) and ethyl ether (c) in the presence of L-AIE-F (2 cm × 2 cm) for different periods of time.

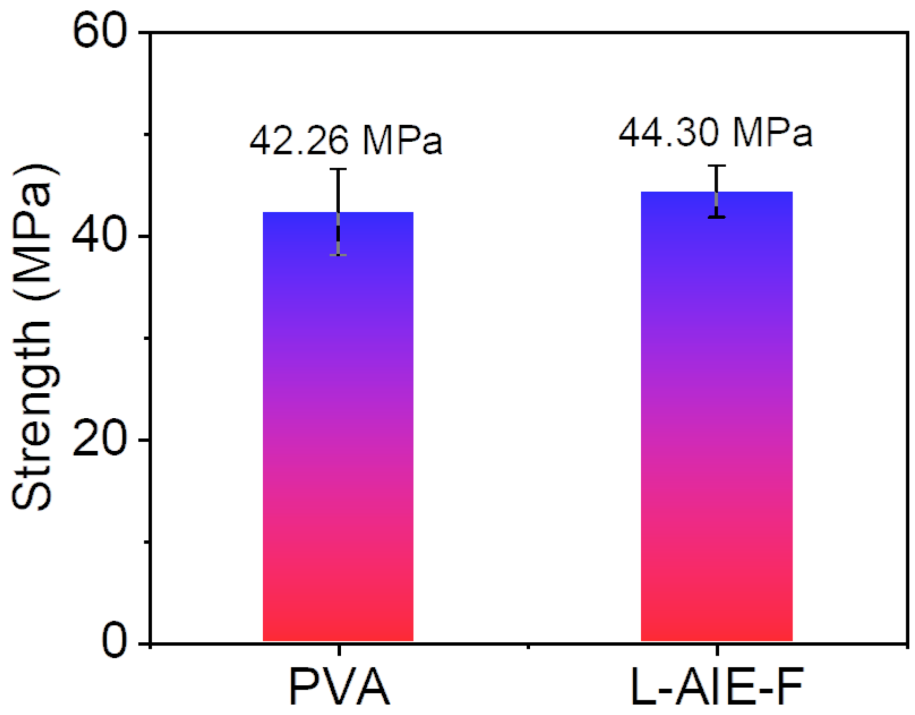

Figure S10. The tensile strength of L-AIE-F and PVA.

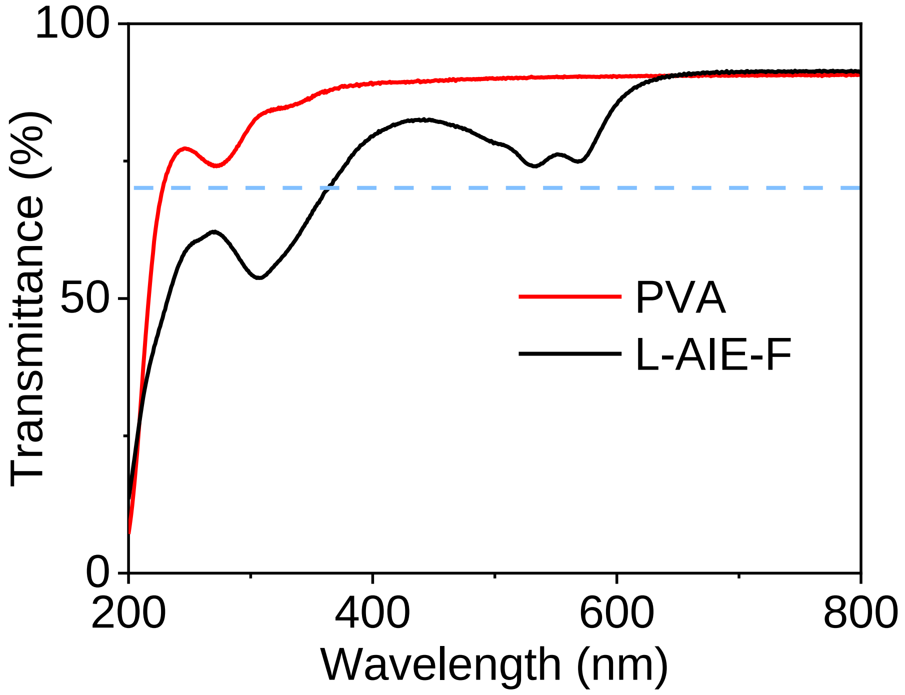

Figure S11. UV-vis light transmittance of PVA and L-AIE-F.

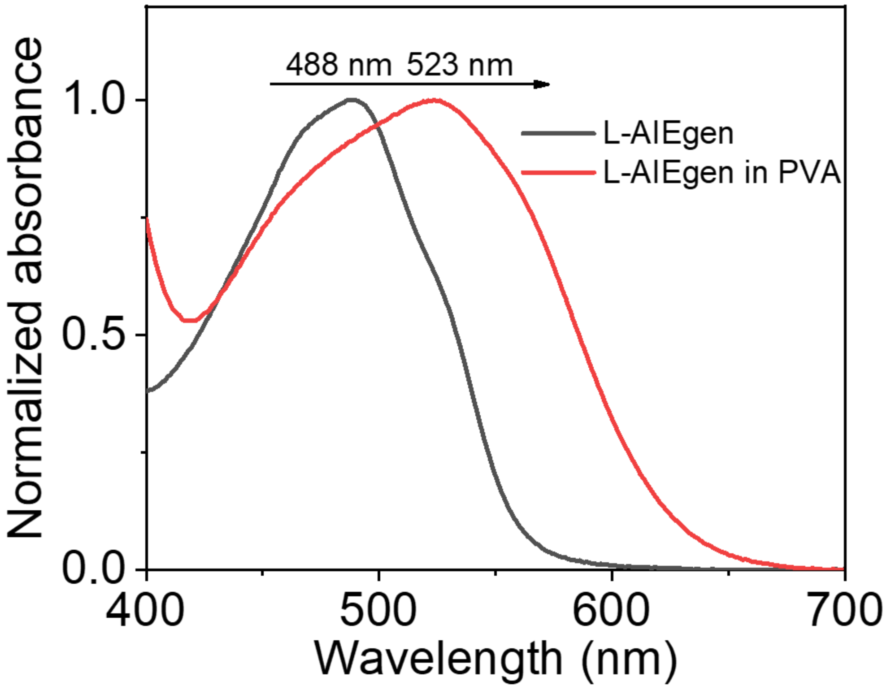

Figure S12. The normalized absorbance of aqueous L-AIEgen and L-AIEgen in aqueous PVA.

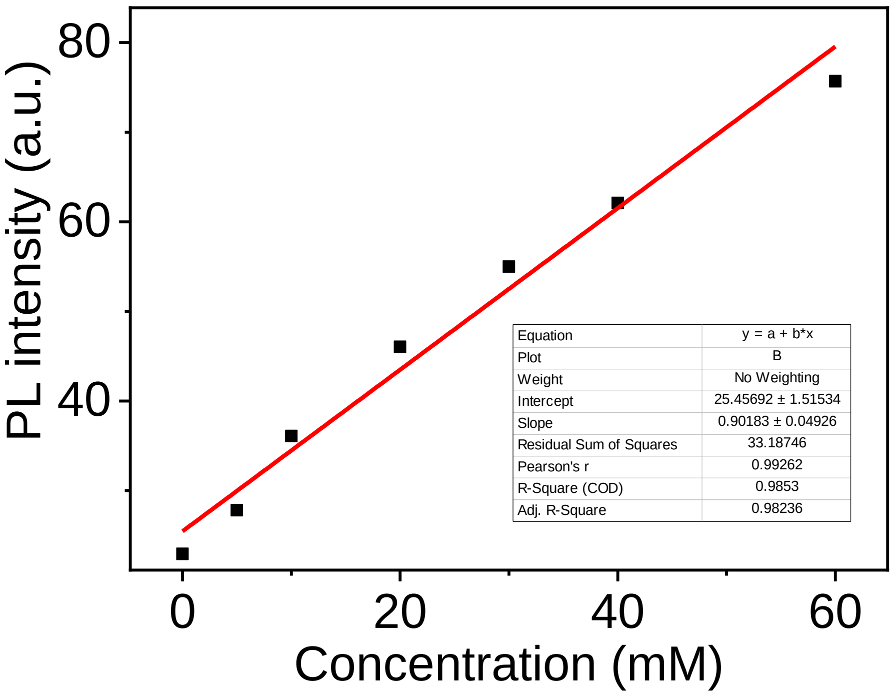

Figure S13. Linear fitting of PL intensity at 645 nm of L-AIE-F *vs* concentration of LX-144 (THF solution).
